# Cobalt Sulfide/Graphene Composite Hydrogel as Electrode for High-Performance Pseudocapacitors

**DOI:** 10.1038/srep21717

**Published:** 2016-02-16

**Authors:** Xiaoqian Meng, Jin Deng, Junwu Zhu, Huiping Bi, Erjun Kan, Xin Wang

**Affiliations:** 1 Key Laboratory for Soft Chemistry and Functional Materials (Nanjing University of Science and Technology), Ministry of Education, Nanjing, 210094, China

## Abstract

Graphene and its composite hydrogels with interconnected three-dimensional (3D) structure have raised continuous attention in energy storage. Herein, we describe a simple hydrothermal strategy to synthesize 3D CoS/graphene composite hydrogel (CGH), which contains the reduction of GO sheets and anchoring of CoS nanoparticles on graphene sheets. The formed special 3D structure endows this composite with high electrochemical performance. Remarkably, the obtained 3D CGH exhibits high specific capacitance (*C*_*s*_) of 564 F g^−1^ at a current density of 1 A g^−1^ (about 1.3 times higher than pure CoS), superior rate capability and high stability. It is worth mentioning that this methodology is readily adaptable to decorating CoS nanoparticles onto graphene sheets and may be extended to the preparation of other pseudocapacitive materials based on graphene hydrogels for electrochemical applications.

Recently, supercapacitors have received noteworthy attention for their importance in energy-efficient, eco-friendly, high-power, and high-energy devices, and these excellent properties make them as one of the most promising candidates for next-generation energy-storage devices^1^,^2^,^3^,^4^,^5^.

Due to its ultra-large specific surface area, good chemical stability, excellent mechanical properties, and extremely high electron mobility, graphene, as a single atom thick two-dimensional (2D) carbon nanostructure material, has attracted tremendous interest in the past years^6^,^7^,^8^,^9^,^10^. Especially, graphene shows a great potential as an electrode material for supercapacitors owing to its outstanding properties such as high surface area and electrical conductivity. The mechanism of energy storage is mainly associated with the electrical double-layer capacitance (EDLC) which stems from the pure electrostatic charge accumulated at the electrode/electrolyte interface. However, suffering from the intersheet van der Waals attractions, the graphene sheets would inevitably restack into face-to-face-stacked multilayered graphene^11^,^12^. Particularly, the design of graphene hydrogels (GH) have been proved to be an effective approach for addressing this issue. The microporous networks endow graphene hydrogel with facile access and diffusion of ions. For example, Zhang *et al*. prepared the 3D TiO_2_-graphene hydrogel, which exhibited improved adsorption capacities and photocatalytic activities^13^. Wu *et al*. demonstrated the synthesis of asymmetric supercapacitor using MnO_2_/rGO hydrogel as the positive electrode and pure rGO hydrogel as the negative electrode, exhibiting a high performance with an energy density of 21.2 Wh kg^−1^ and power density of 0.82 kW kg^−1^ 14.

Cobalt sulfides (CoS, CoS_2_ and CoS_x_) have been widely used as electrode materials for pseudocapacitors as with other metal sulfides (NiS, MoS_2_)^15^,^16^,^17^,^18^,^19^,^20^. Many recent studies have proved that cobalt sulfides show a great potential as an electrode material for supercapacitors. Notably, the cobalt sulfides with various nanostructures exhibit the excellent electrochemical performances. For example, Luo and Ray *et al*. prepared the three-dimensional flower-like CoS hierarchitectures and CoS layer on titania nanotubes for application of high performance supercapacitor, respectively^21^,^22^. However, these materials are still plagued with problems of rapid capacity fading and low rate performances due to their pulverization during the charge and discharge cycles. Given the special 3D structure of graphene hydrogels, the combination of cobalt sulfides and graphene hydrogels might be an effective approach to improve their electrochemical performance^23^,^24^.

Herein, we demonstrate a facile one-step approach for the fabrication of macroscopic cobalt sulfide/graphene hydrogels with 3D structure. In this procedure, the self-assembly of GO sheets and *in situ* deposition of cobalt sulfide nanoparticles on GO sheets occurred simultaneously, resulting in the formation of CGH. Interesting, the weight ratio of feeding Co(CH_3_COO)_2_·4H_2_O to GO greatly affects the structure of obtained CGH composite. The presence of excess cobalt salt in the reaction mixture would lead to the collapse of 3D hydrogel structure, which suggests that a proper concentration of GO is necessary for the formation of CGH. Furthermore, the as-prepared CGH exhibits high capacitance performance and excellent rate capability. This method is expected to be applied in the preparation of other metal sulfides-graphene composites for high-performance energy storage materials. Meanwhile, the combination of GH with pesudocapacitive materials opens up a promising new strategy for the application of graphene-based materials to supercapacitor designs.

## Results and Discussion

**Figure 1:**
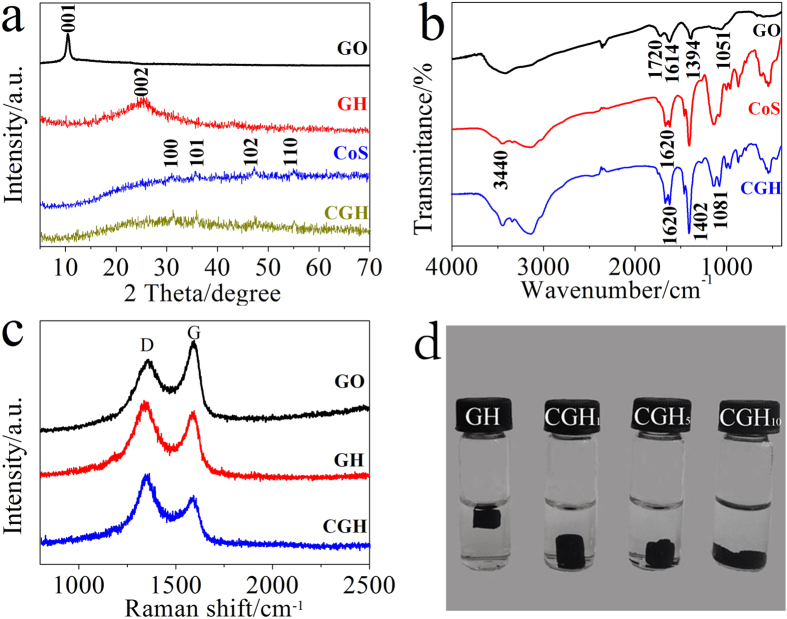
(**a**) XRD patterns of GO, GH, CoS and CGH composites. (**b**) FT-IR spectra of GO, CoS and CGH. (**c**) Raman spectra of GO, GH and CGH composites. (**d**) Photographs of different weight ratio of CGH.

As shown in Fig. 1a, the XRD pattern of GO shows an intensive peak at around of 2θ = 10.4° corresponding to the (001) reflection of GO^25^. In contrast to GO, after hydrothermal reduction, the freeze dried GH exhibits a broad peak centered at 2θ* = *25°, which is attributed to unordered restacking of reduced GO sheets. For the pure CoS and CGH, all diffraction peaks can be indexed to a hexagonal phase of CoS with the lattice parameters of a = b = 3.377 Å and c = 5.15 Å (JCPDS No.75-0605)^21^,^26^. In addition, it is worth mentioning that no typical patterns of GH (002) or GO (001) were observed in CGH. Actually, the unordered restacking of graphene sheets existed in 3D hydrogel would make their diffraction peaks weaken or even disappear. Moreover, the *in-situ* formed CoS nanoparticles might attach onto GH sheets and prevent their aggregation and restacking, which also could weaken the peak intensity of GH.

To investigate the deoxygenation of GO, the obtained samples were subjected to FT-IR measurement (Fig. 1b). For CGH, the disappearance of almost all the characteristic peaks of GO, including C = O stretching vibrations of carboxyl groups (1720 cm^−1^), the stretching vibrations of carbon backbone (1614 cm^−1^), O-H deformation vibrations of tertiary C-OH groups (1394 cm^−1^), and C-O stretching vibrations of epoxy groups (1051 cm^−1^), indicates the successful reduction of GO. The -OH stretching vibrations in the region of ~ 3440 cm^−1^ are attributed to the hydroxyl and carboxyl groups of GH and residual water between GH sheets^7^. In addition, the peaks at 1081 and 1620 cm^−1^ correspond to the bending vibration of sulfonated groups and bending vibration of absorbed H_2_O in pure CoS and CGH^27^. The peaks of CGH in the range 400–1000 cm^−1^ are in accord with pure CoS, confirming the presence of CoS in CGH. Moreover, the peaks at 536 and 628 cm^−1^ correspond to the vibrations of N-C-N and C = S in thiourea, while 453, 859, and 958 cm^−1^ correspond to N-C = S^28^,^29^. Compared to pure CoS, the slight shifts and broadening of these peaks indicated the possible formation of a covalent interaction after the *in-situ* deposition of CoS on graphene^30^.

The compositions of GO, GH and CGH were further investigated by Raman spectra (Fig. 1c). It has been reported that Raman peaks of G and D bands would shift to lower values when GO was reduced to graphene^31^. As we know, the G band (at ~1589 cm^−1^) is associated with bond stretching of the sp^2^ carbon pairs in both rings and chains, while the D band (at ~1352 cm^−1^) is due to the breathing mode of aromatic rings with dangling bonds in plane terminations^32^,^33^,^34^. So the intensity ratio of D to G band is usually employed to determine the degree of disorder for carbon materials^5^. It is found that the Raman spectra of GH and CGH show an increased D/G intensity ratio compared to GO. Notably, the CGH presents a higher D/G intensity ratio (1.41) compared to those of GO (0.83) and GH (1.15), indicating that lower disorders and defects of graphene sheets in CGH, and that GO was reduced to some extent during the formation of composite at high temperature. This result could be attributed to the declined restacking degree of graphene sheets after the introduction of CoS nanoparticles, which matches very well with the XRD results. In our experiment, CGH with well-defined 3D gel-like cylinder can be formed under different weight ratios (denoted by r) of feeding Co(CH_3_COO)_2_·4H_2_O/GO (Fig. 1d). Clearly, with the elevated r value, the gel-like structure of CGH gradually loses its perfect shape of cylinder caused by the increased loading content of CoS nanoparticles (Fig. S2g,h).

**Figure 2:**
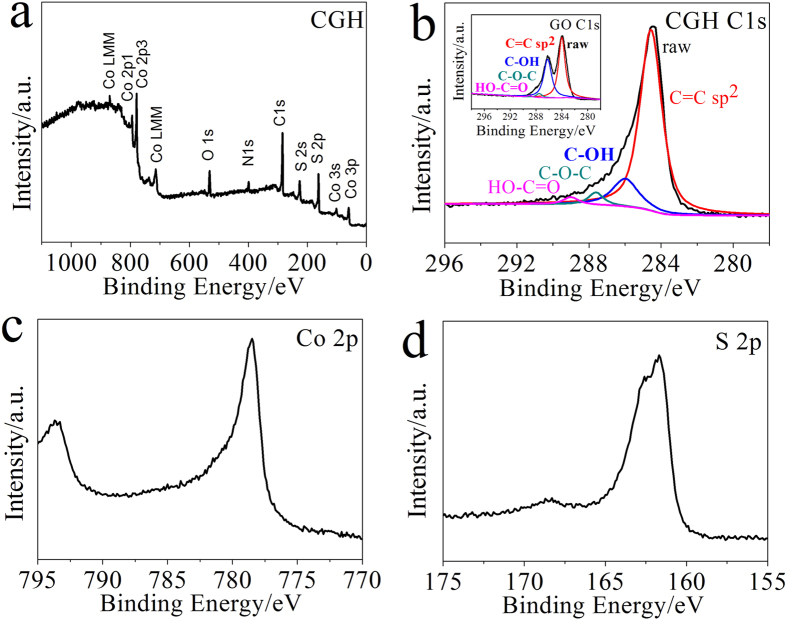
XPS spectra of freeze-dried samples. Wide (**a**) and deconvoluted (**b**) XPS spectra of the as-prepared CGH, the inset of (**b**) is C 1s spectrum of GO, (**c,d**) Co 2p and S 2p spectra of CGH.

XPS has been proved to be a useful tool for identifying the oxidation state of elements. As shown in Fig. 2a, the CGH consists of Co, S, C and O as the predominant elements. In Fig. 2b, the deconvoluted XPS peaks of C1s centered at the binding energies of 289, 287.5, 286.2, and 284 eV were assigned to the C = O, C-O-C, C-OH and C = C, respectively. It can be clearly seen that the most carbon atoms were sp^2^ hybridized, and the intensity of oxygenated functional groups (HO-C = O, C-O-C, and C-OH) on graphene sheets in CGH and GH (Fig. 2b, Fig. S1) were obviously decreased compared with those of GO, which further demonstrates the occurrence of high degree deoxygenation of GO during the hydrothermal process. The Co 2p spectrum shown in Fig. 2c presents a main peak at 778.6 eV with a shoulder peak at 793.7 eV, indicating the presence of Co^2+^ oxidation state^12^. The peaks at 161.7 and 168.6 eV in Fig. 2d could be assigned to the spin-orbit couple of S 2p_3/2_ and S 2p_1/2_, respectively. The S 2p peak at 161–163 eV suggests that the S species exist as S^2−^ in the composite, corresponding to the binding energy of Co-S^17^. So we can confirm the Co^2+^ oxidation state and the dominating composition as CoS, which was consistent with the above XRD measurement.

**Figure 3:**
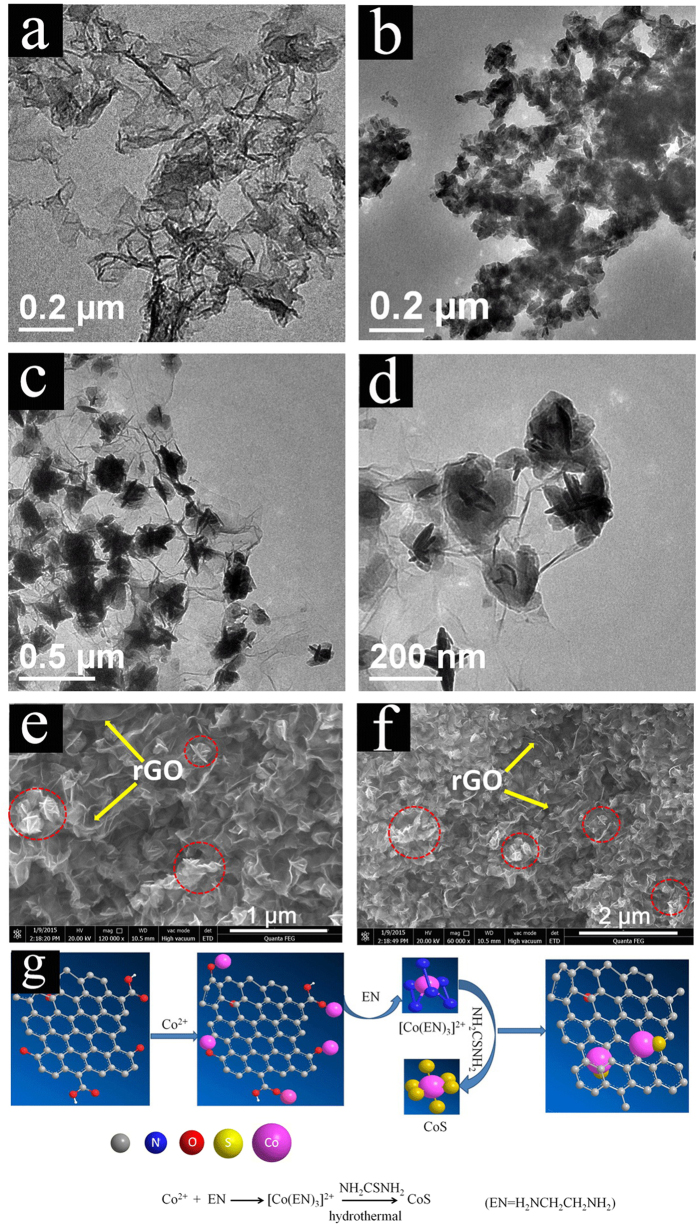
TEM images of the (**a**) GH (**b**) CoS (**c,d**) CGH composites and (**e,f**) FESEM images of CGH; (**g**) schematic illustration of formation mechanism of the CGH.

With the aid of hydrothermal reduction process at high temperature, the graphene sheets self-assembled tightly to form GH with 3D interconnected network structure (Fig. 3a, Fig. S2a,b). Figure 3b and Fig. S2c,d reveal that the CoS is actually composed of spindle particles with severe aggregation. Interestingly, there is a slight change in morphology for CoS between pure CoS and CGH. Obviously, the flower-like CoS particles with improved dispersity can be observed in the presence of graphene (Fig. 3c,d, Fig. S2e,f), which indicates that there is a different crystals growth process with or without graphene supports. FESEM images (Fig. 3e,f) show that the as-obtained CGH is composed of well-defined 3D plicate network with micrometer interconnected pores, in which the flower-like CoS nanoparticles were densely anchored onto the surface of graphene sheets (the CoS nanoparticles were marked by the red circles and the GH sheets were labelled by the white arrows in Fig. 3e,f). With the increased loading contents of CoS, the surface of graphene sheets is covered almost completely by CoS nanoparticles (CGH_r=10_, Fig. S2g,h). Actually, the pores existed in 3D structure would be occupied by CoS particles with the increased loading contents of CoS, which is not conducive to the diffusion and migration of electrolyte ions.

For the growth of flower-like CoS nanoparticles using thiourea as precursor, a possible mechanism has been proposed to explain the nucleation and growth as follows:^22^


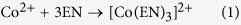



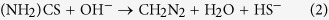



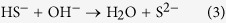



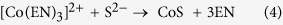
Firstly, the Co^2+^ ions tended to adsorb onto surfaces of negative GO sheets *via* the electrostatic interactions, which then would react with ethanediamine (EN) to produce [Co(EN)_3_]^2+^ ions. Meanwhile, S^2−^ could be released gradually, as the decomposition product of thiourea with increased temperatures. Then, [Co(EN)_3_]^2+^ further reacted with the generating S^2−^ ions to form CoS nucleus, as shown in Fig. 3g. As a result, the CoS crystalline were formed and grew gradually. In this processes, the thiourea played an important role: on the one hand, it induced the decrease of concentration of the free Co^2+^ ion; on the other hand, it reduced the rate of crystal growth. Moreover, the existence of EN effectively restrains the agglomeration of fine nanoparticles with high energy. This is favorable for the formation of CoS nanocrystals. Afterwards, the formed nucleus gradually grew into flower-like nanoparticles and *in situ* deposited on both sides of graphene sheets. At the same time, GO was reduced in the hydrothermal process under high temperature and self-assembled to form the final composite hydrogel (CGH) with cross-linked 3D network, owing to *π-π* stacking interaction of conjugated RGO sheets.

To explore the electrochemical properties of as-synthesized nanocomposites, the samples were fabricated into supercapacitor electrodes and characterized with cyclic voltammograms (CVs) and galvanostatic charge/discharge measurements. CV response of as-synthesized samples was carried out at a scan rate of 50 mV s^−1^ in the potential range of −0.5–0.5 V using 6 M KOH as electrolyte. As we know, the specific capacitance is proportional to the area under the CV curve, and we can find that the capacitance of CGH is clearly higher than that of pure CoS. Notably, two typical redox couples can be observed in the CV curves, demonstrating the pseudocapacitive characteristics of the as-prepared CoS and CGH electrode. According to the literatures^16^,^21^,^22^, the two anodic peaks are likely due to the oxidations of CoS to CoSOH and CoSOH to CoSO, and two plausible reactions are proposed for electrochemical reactions.


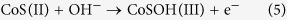



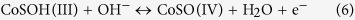


**Figure 4:**
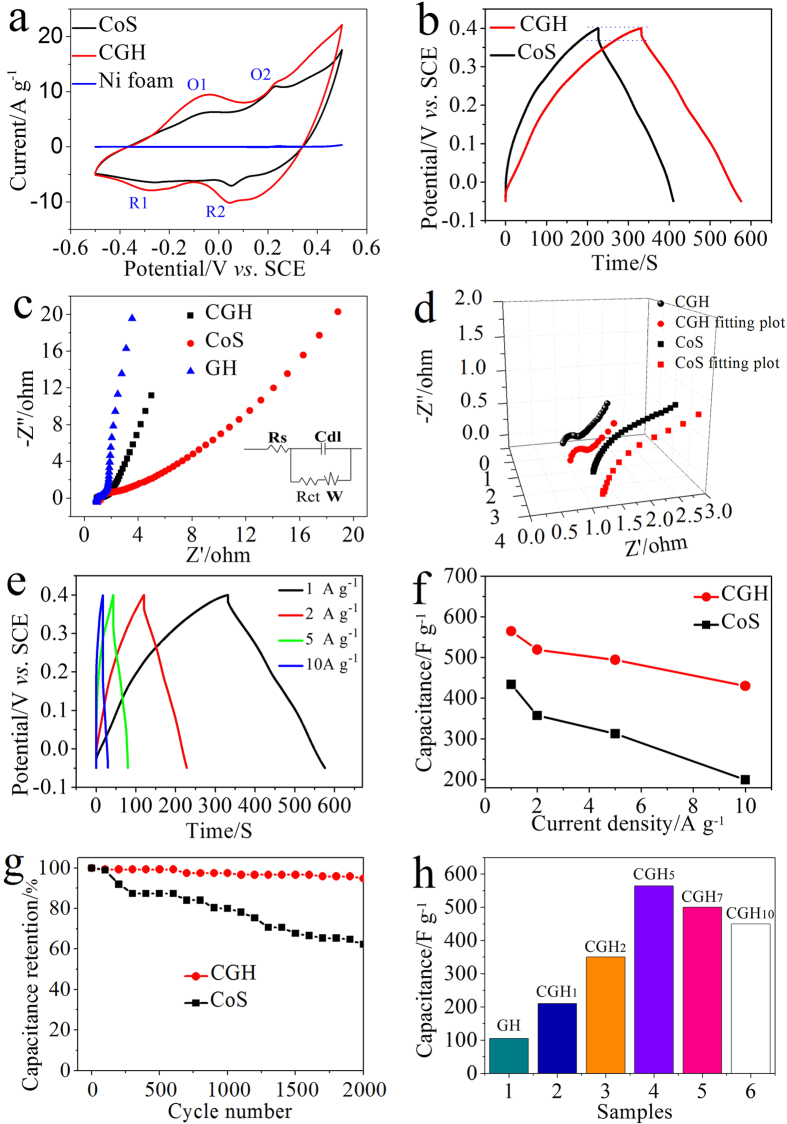
(**a**) CV curves of Ni foam, freeze-dried CoS, and CGH at a scan rate of 50 mV s^−1^; (**b**) galvonostatic charge-discharge curves of freeze-dried CoS and CGH at the current density of 1 A g^−1^; (**c**) Nyquist plots of freeze-dried CoS, GH, and CGH, the inset shows the corresponding Randles circuit model; (**d**) Nyquist plots of CoS and CGH, and their curves after fitting with an equivalent electrical circuit (**e**) galvonostatic charge-discharge curves of CGH at different current densities; (**f**) Gravimetric capacitances measured at various charge/discharge current densities (**g**) The cycling performance at the current density of 1 A g^−1^. (**h**) *C*_*s*_ of CGH with different weight ratios of Co(CH_3_COO)_2_·4H_2_O to GO.

CV of pure Ni foam was tested at a scan rate of 50 mV s^−1^ in the potential range of −0.5–0.5 V (Fig. 4a). Obviously, the area under CV curve is extremely small for the naked nickel foam, suggesting that the *C*_*s*_ of nickel foam itself is very little and negligible, which further demonstrates that the main component of the measured *C*_*s*_ is produced from the pseudocapacitive redox process of CGH. Figure 4b shows the galvanostatic discharge curves at the current density of 1 A·g^−1^. The *C*_*s*_ is calculated according to *Cs* = *I*·Δ*t*/(Δ*V*·*m*) from the discharge curves, where *I* (A) is the constant discharge current, m (g) is the mass of active electrode material, Δ*t* (s) is the discharge time, and Δ*V* (V) is the potential drop during discharge. Accordingly, the calculated *C*_*s*_ values of CGH and CoS are 564 F g^−1^ and 433 F g^−1^, respectively. Obviously, the CGH presents higher *C*_*s*_ value than that of its counterparts, indicating the beneficial synergistic effect between CoS and GH, which is coincident with the CVs results.

In addition, the symmetric supercapacitor cell was assembled to measure the performances of CGH and CoS, using CR 2032-type coin cells. In full cell tests (Fig. S5), the CV and galvonostatic charge-discharge curves were presented. Seen from the results, both the area under the CV curve and the discharge time of CGH indicate higher capacitance than that of CoS, which further demonstrate the more superior performances of CGH than CoS.

To further understand the electrochemical performance characteristics, the electrochemical impedance spectroscopy (EIS) was carried out in the frequency range of 10000 Hz to 0.01 Hz at the open circuit potential by applying an AC voltage with 5 mV amplitude (Fig. 4c,d). These EIS data were fitted based on an equivalent circuit model consisting of bulk solution resistance (Rs), charge-transfer resistance (Rct), double-layer capacitance (Cdl), and Warburg resistance (W)^3^,^23^,^35^. Generally, the Rs is a representative of bulk resistance, which is a combination of pore electrolyte resistance, bulk electrolyte resistance, and contact resistance between the current collector and electroactive material. Rs is represented as the real axis intercept of the impedance plot, and the Rs of GH, CGH, and CoS were calculated to be 0.94, 0.99, and 1.22 ohm, respectively. The lower Rs value presented by CGH compared to CoS may attribute to the 3D plicate network introduced by graphene, which allows efficient accessibility for electrolyte to electrode surface by shortening the ion diffusion pathway. In addition, the EIS data (Fig. 4d) reveals that the CGH nanocomposite electrodes possesses a medial Rct (0.21 ohm) between GH (0.05 ohm) and CoS (0.91 ohm), which is much smaller compared with that of CoS. Actually, the Rct is a representative of electrochemical reaction resistance, also known as faradaic resistance, which determines the response rate of electrode material in electrolyte. The low Rct reveals that CGH has much higher electric conductivity comparing with pure CoS. The linear part after the semicircle involves the diffusion process of ions, and the higher slope of CGH demonstrates that it owns faster ion transfer rates between electrode and electrolyte than pure CoS. The results clearly demonstrate that the CGH has favorable charge-transfer kinetics and fast ions transport rates, thus exhibiting the enhanced pseudocapacitive performance owing to its 3D structure and the beneficial synergistic effects between CoS and graphene.

Furthermore, the rate capability is another important parameter related to electrochemical capacitors for assessing their application potential. It is noticed that the CGH composite can offer specific capacitance of 564, 519, 496 and 430 F g^−1^ at 1, 2, 5 and 10 A g^−1^, respectively. The CGH shows 76.2% retention of *C*_*s*_ with the increasing current density from 1 to 10 A g^−1^, but the value of pure CoS is merely 57.7% (Fig. 4e,f, Fig. S3a,b). Remarkably, the CGH-based electrode exhibits excellent electrochemical stability with 94.8% retention of *C*_*s*_ over 2000 cycles, which is much higher than that of pure CoS (62.2% retention) (Fig. 4g).

In addition, the weight ratios (r) of Co(CH_3_COO)_2_·4H_2_O to GO have remarkable effects on its electrochemical properties. As shown in Fig. 4h, the *C*_*s*_ initially increases with the elevated r, but declines when the r further increases over 5. Acturally, as we discussed earlier, the pores of 3D structure would be blocked when the excess CoS particles were introduced, which would hinder the diffusion of electrolyte ions (Fig. S3c,d). Meanwhile, the excess CoS particles on surface of graphene sheets would inevitably aggregate and result in the decline of condutivity of obtained CGH composite. The combination of the above several factors leads to the decrease of *C*_*s*_. In contrast to pure CoS, the improved specific capacitance at high current density and better cycle performance presented by CGH can be attributed to the special 3D structure, excellent conductivity of graphene sheets and beneficial synergistic effects between CoS and graphene.

To sum up, the attractive electrochemical performance exhibited by CGH could be attributed to the directly grown of CoS nanoparticles on graphene sheets, including a synergistic effect from the two components. Notably, CGH composite in our experiment with 3D interconnected network structure inherited both the flower-like CoS and the 3D framework gelated from graphene. The unique nanostructure of CGH effectively prevents the restacking of graphene sheets, and keeps good adhesion and electrical contact between CoS and graphene sheets. Therefore, the improved capacity value, cycling performance, and rate capability of CGH could be ascribed to the inheritance of the excellent properties of graphene hydrogels system.

## Conclusions

In this work, we have developed a hydrothermal process for the synthesis of CGH composite with the unique 3D microcosmic structure. The special strowucture not only prevents the CoS nanoparticles from aggregating, but also facilitates the electrolyte ions transferring in this special space structure, leading to the improved electrochemical properties. This method provides a considerable approach to the design and fabrication of novel 3D graphene-based hydrogel pseudocapacitive electrode materials with enhanced electrochemical properties.

## Methods

### Synthesis of CGH

All chemical reagents were obtained from Sinopharm Chemical Reagent Company (China). GO was prepared from graphite powders according to a modified Hummers’ method, as previously reported^36^,^37^. Then we preprocessed the prepared GO by a base wash to get the homogeneous GO^38^. The CGH hydrogel composites with different mass ratios of CoS/G (graphene) were synthesized. Herein, the mass of CoS was controlled by changing the feeding ratios of Co(CH_3_COO)_2_·4H_2_O to GO. A typical route for the synthesis of CGH composite with the weight ratio of feeding Co(CH_3_COO)_2_·4H_2_O to GO of 5:1, is as follows. 1 mmol (249 mg) of Co(CH_3_COO)_2_·4H_2_O and 2 mmol (152 mg) of thiourea were dissolved in 19 mL of NaOH-washed GO dispersion (2.6 mg mL^−1^), then 300 uL of ethanediamine was added into the above mixture. After vigorous stirring for 1 h, the copies of formed 1 mL suspension were packed into several glass bottles, and transferred into a Teflon-lined stainless steel autoclave. It was sealed and maintained at 180 °C for 12 h followed by cooling down to room temperature. The prepared hydrogels were carefully taken out, immersed into water and washed with water for several times to remove the residual ions. The obtained hydrogel is defined as CGH, and it was freeze dried for the further characterization (Fig. S4).

### Characterization of materials

Powder X-ray diffraction (XRD) analyses were performed on a Bruker D8 Advance diffractometer with Cu Kα radiation (λ = 1.5418 Å). Raman spectra were recorded with a Renishaw inVia Reflex Raman microscope with a solid-state laser (excitation at 514 nm, 20 mW) at room temperature. The beam diameter was approximately 1 μm on the sample surface. FTIR spectra of KBr powder pressed pellets were recorded on a FTIR-8400S spectrometer. Morphologies of as-obtained products were observed on a transmission electron microscope (TEM, JEOL JEM-2100) and scanning electron microscope (SEM, JEOL JSM-7001F). X-ray photoelectron spectra (XPS) were recorded on a PHI QUANTERA II X-ray photoelectron spectrometer, using monochromatic Al Kα radiation as the exciting source (energy resolution <0.60 eV).

### Electrochemical characterization

The electrochemical measurements were carried out in a three-electrode electrochemical cell containing 6 M KOH aqueous solution as the electrolyte. The working electrode was prepared by mixing 80 wt% of freeze-dried CGH nanocomposite, 10 wt% of acetylene black and 10 wt% polytetrafluoroethylene (PTFE) binder. The mixture was then pressed onto the Ni foam electrode and dried at 60 °C in a vacuum over for 10 h. Platinum foil and a saturated calomel electrode (SCE) were used as the counter and reference electrodes, respectively. The CV, galvanostatic charge-discharge and EIS measurements were conducted on a CHI 760 D electrochemical workstation (Shanghai CH Instrument Company, China). The details for the preparation process of electrodes using a two-electrode system were provided in the Supporting information (Section 3, Supporting information).

## Additional Information

**How to cite this article**: Meng, X. *et al*. Cobalt Sulfide/Graphene Composite Hydrogel as Electrode for High-Performance Pseudocapacitors. *Sci. Rep.* **6**, 21717; doi: 10.1038/srep21717 (2016).

## Supplementary Material

Supplementary Information
