# Supplementary material for: Cobalt Sulfide/Graphene Composite Hydrogel as Electrode for High-Performance Pseudocapacitors
Source: Sci Rep. 2016 Feb 16;6:21717. doi: 10.1038/srep21717 (PMC4754945; doi:10.1038/srep21717)
Supplement: Supplementary Information [file srep21717-s1.doc]

**Supporting Information**

**Cobalt Sulfide Coated On Special Structure Graphene For High-Performance Pseudocapacitors**

Xiaoqian Meng, Jin Deng, Junwu Zhu,* Huiping Bi, Erjun Kan, Xin Wang

**Section 1**

**
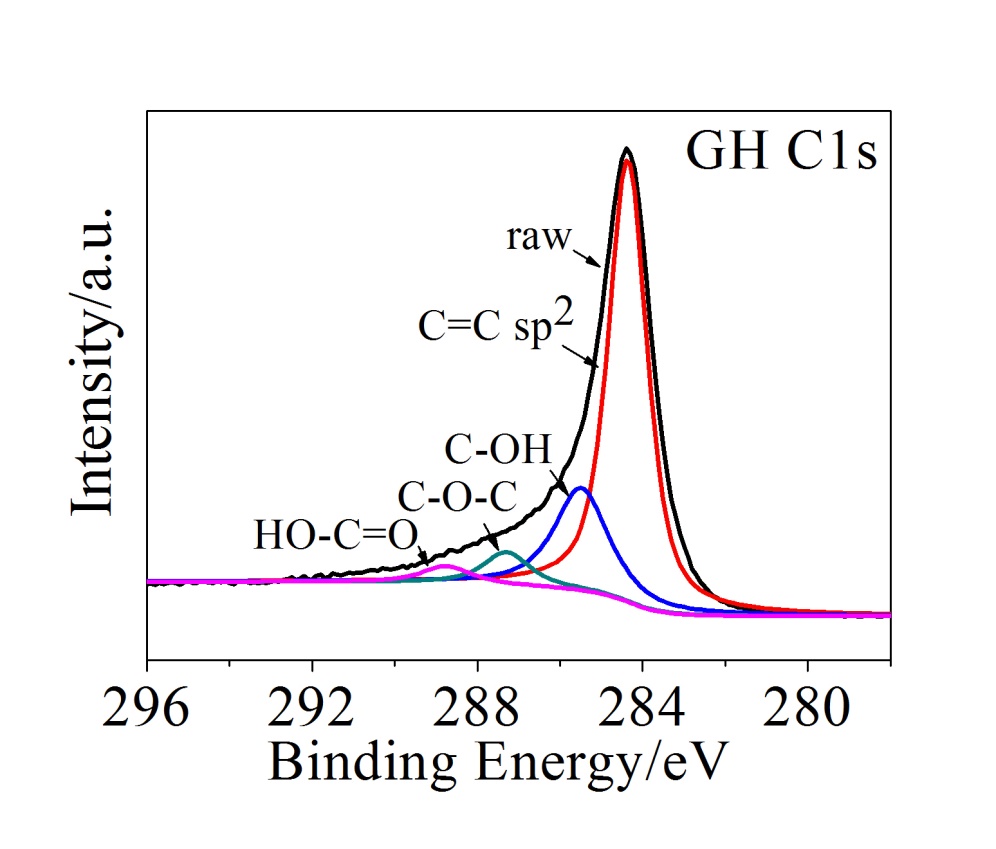
**

**Figure S1.** C1s XPS spectra of GH


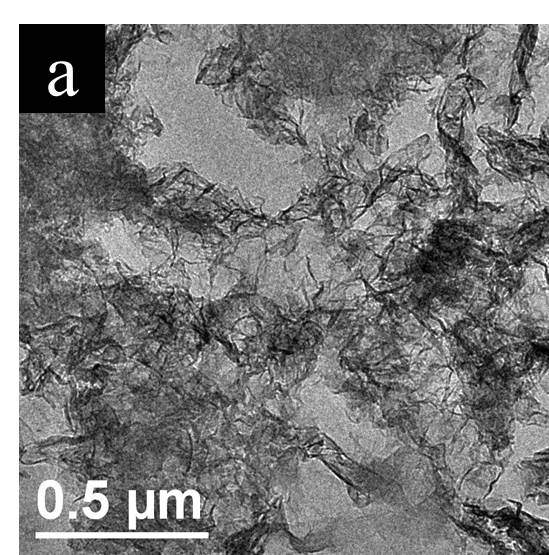

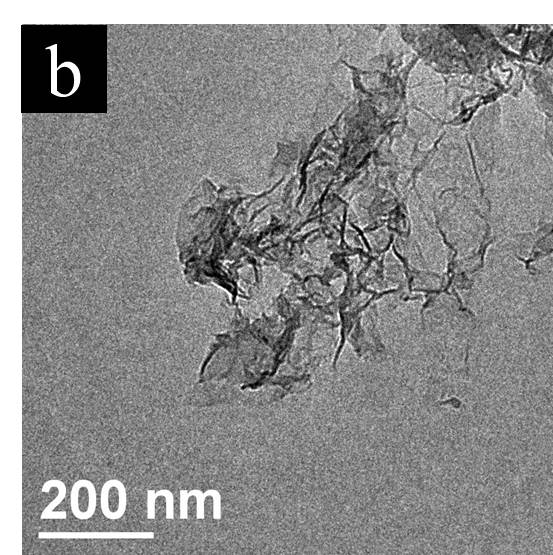

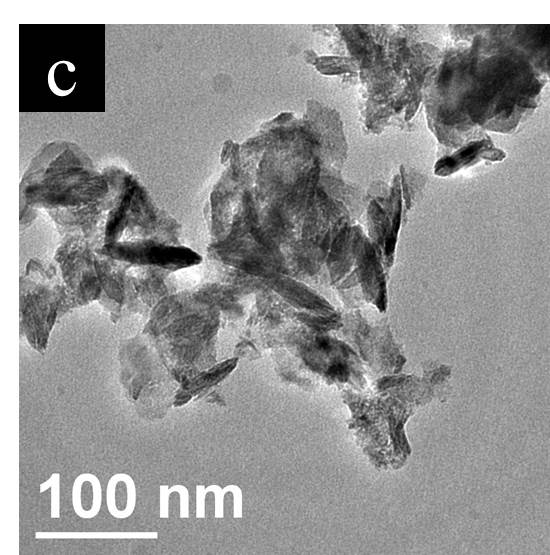

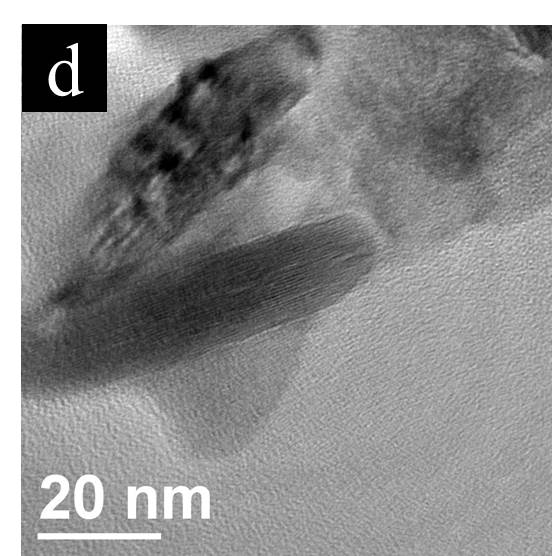

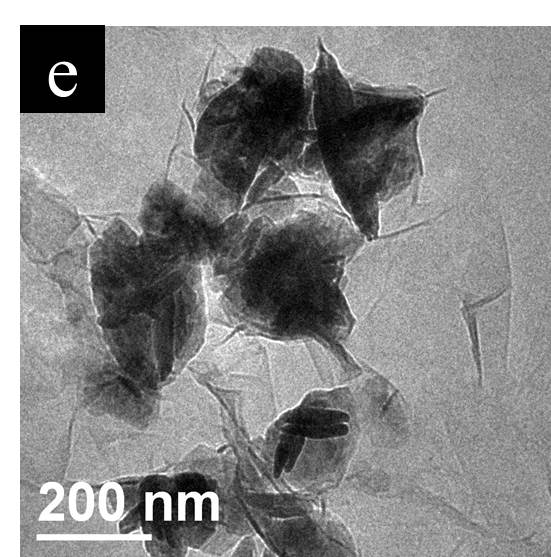

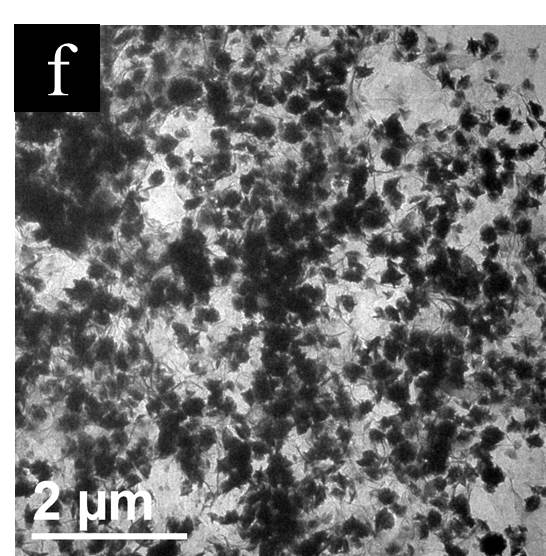

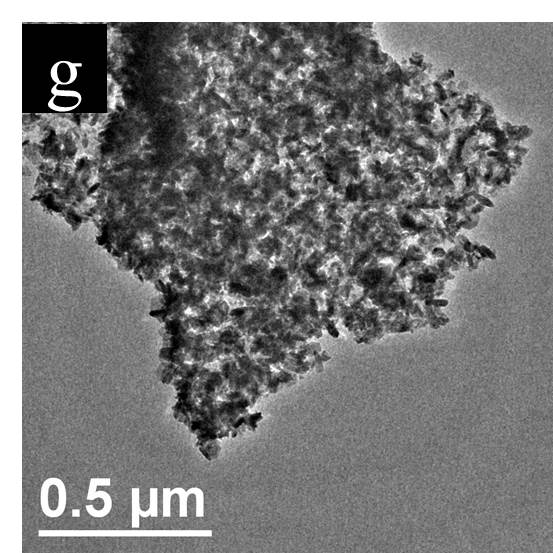

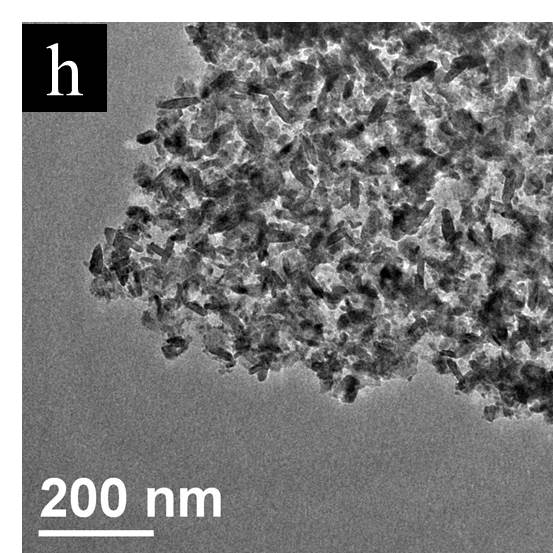


**Figure S2.** TEM images of the (a-b) GH (c-d) CoS (e-f) CGH_r=5_ (g-h) CGH_r=10_ composites.
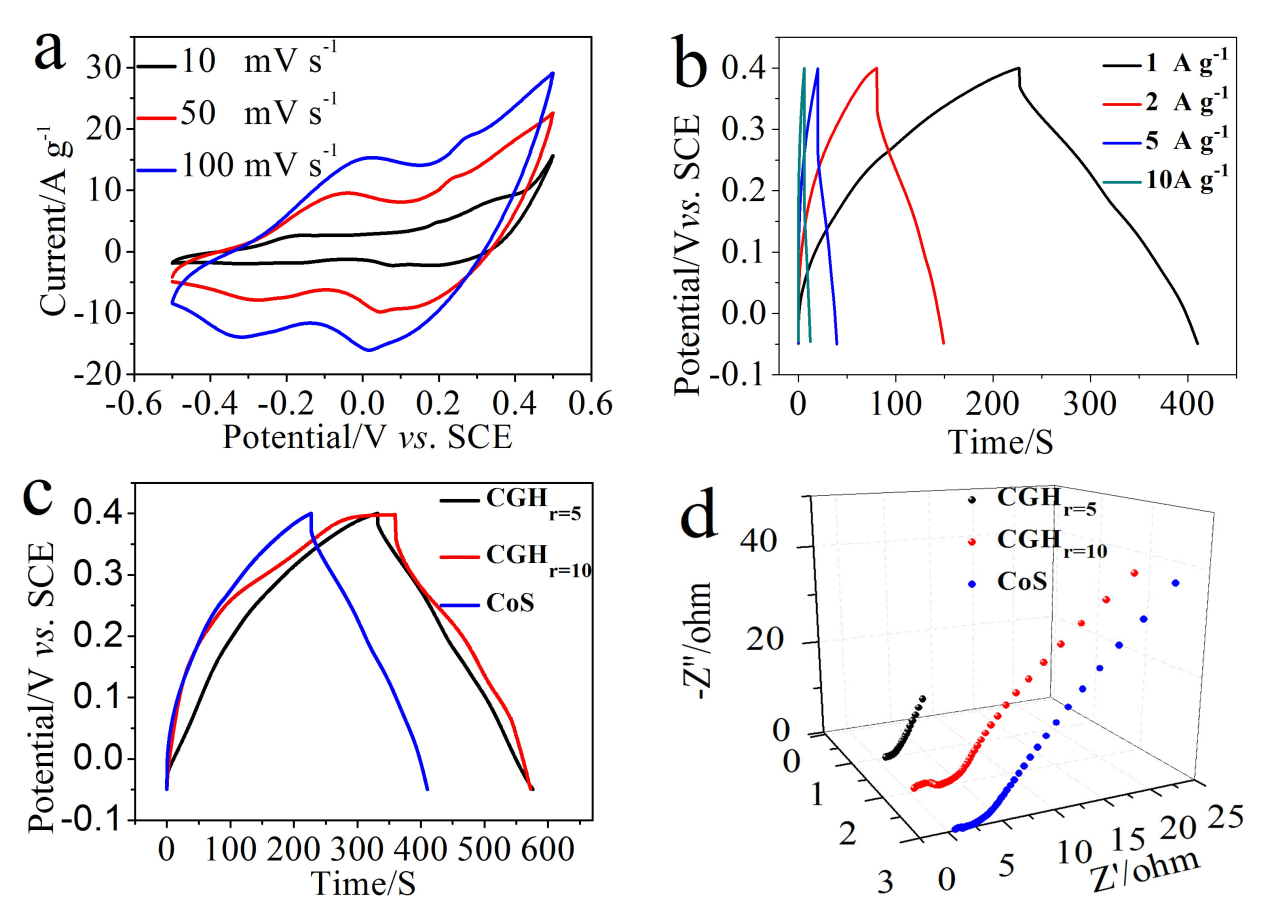


**Figure S3.** (a) CV curves of freeze-dried CGH; (b) galvonostatic charge-discharge curves of freeze-dried CoS at different densities (c) galvonostatic charge-discharge curves of CGH _r=5_，CGH _r=10_ and CoS at 1 A·g^−1^ (d) Nyquist plots of CGH _r=5_, CGH _r=10_ and CoS

**Section 2**

GO prepared by modified Hummers’ method (200 mg) was dispersed in H_2_O (200 ml) to form a stable light brown solution. NaOH (0.2 g) was added into the above solution under constant stirring. The solution becomes dark immediately when the NaOH was added, which was then heated to 70 °C for 1 h. The resultant dark brown solution was centrifuged (10000 rpm, 20 min) leaving a dark brown solid and light brown supernatant. The obtained dark brown product was washed with deionized water and centrifuged. The obtained solid was protonated with dilute HCl (250 mL, 0.02 M) and stirred for 1 h at 70 °C. Once cooled to room temperature, the solid was collected as before and washed with deionized water. Then the product was freeze-dried and dispersed in water to form a solution (2.6 mg/L).^1^


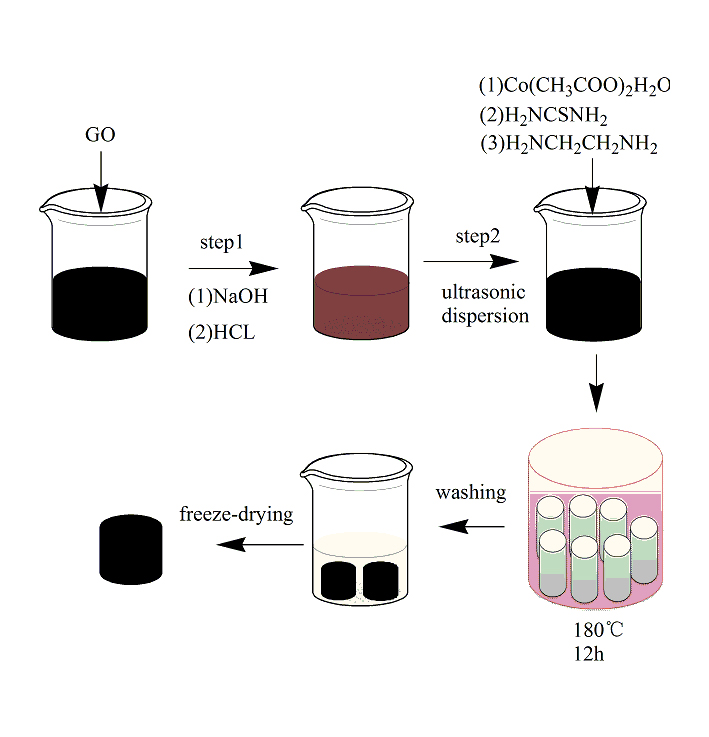


**Figure S4.** schematic diagram to illustrate the preparation process of the CGH composites

**Section 3. Electrochemical measurements with a two-electrode system**

**
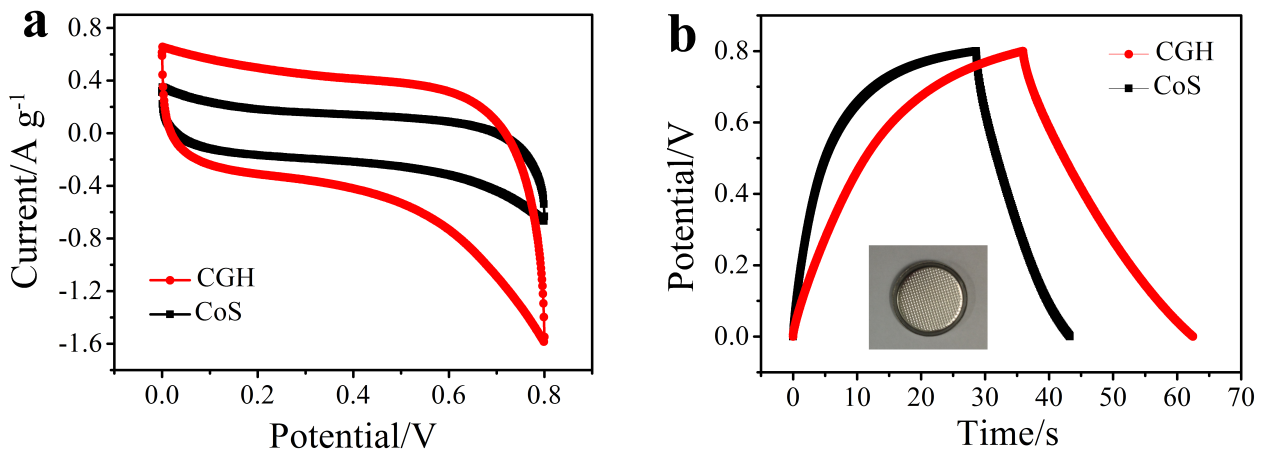
**

**Figure S5.** (a) CV curves of CGH and CoS at 5 mV s^-1^; (b) galvonostatic charge-discharge curves of CGH and CoS at 0.5 A g^-1^, the inset is the photograph of a typical coin cell.

Every working electrode was fabricated by mixing the prepared samples with 5.3 mg of active materials and 1 mg of acetylene black, adding 33 μL (1 wt%) of polytetrafluoroethylene (PTFE) binder to produce a homogeneous paste. The paste was directly pressed onto Ni foam and dried at 60 ^o^C for 12 h. In full cell tests, the symmetric supercapacitor was assembled using CR 2032-type coin cells to measure the device performances (inset of Figure S5b). The electrodes were first activated by charge-discharge curves using a battery test system (LAND CT2001A). Afterwards, the cells were then connected to a CHI760D electrochemical workstation. The CV responses of CGH and CoS were carried out at a scan rate of 50 mV s^-1^ in the potential range of 0-0.8 V. The galvanostatic charge/discharge tests were carried out at a current density of 0.5 A g^-1^. The specific capacitance was calculated according to the equations reported previously.^2,3^

**References**

1. Thomas, H. R. *et al*. Deoxygenation of Graphene Oxide: Reduction or Cleaning? *Chem. Mater.*, **25**, 3580-3588, (2013).
2. Yang, X., Zhu, J., Qiu, L., & Li, D. Bioinspired effective prevention of restacking in multilayered graphene films: towards the next generation of high-performance supercapacitors. *Adv. Mater.*, **25**, 2833-2838, (2011).
3. Chen, H. *et al*. In situ growth of NiCo_2_S_4_ nanotube arrays on Ni foam for supercapacitors: Maximizing utilization efficiency at high mass loading to achieve ultrahigh areal pseudocapacitance. *J. Power Sources*, **15**, 249–257, (2014)
